# Responsibilities of General Practitioners Regarding Diabetes Mellitus During the Month of Ramadan

**DOI:** 10.7759/cureus.55977

**Published:** 2024-03-11

**Authors:** Murad Ali

**Affiliations:** 1 Medicine, Bacha Khan Medical College, Mardan, PAK

**Keywords:** responsibilities, ramadan fasting, general practitioners, diabetes mellitus, ramadan

## Abstract

Muslims practice fasting during the ''holy month of Ramadan'', which poses special difficulties for those who have diabetes. Studies show that a sizable fraction of people with type 1 and type 2 diabetes fast despite the health hazards that come with the condition. This indicates that the incidence of diabetes among Muslims who fast is noteworthy. An increased vulnerability to acute problems, such as hypoglycemia, hyperglycemia, and diabetic ketoacidosis, is caused by extended fasting periods, irregular eating and sleeping routines, and changes in medication regimens. Healthcare professionals (HCPs), especially General Practitioners (GPs), should be involved in advising patients on safe fasting practices to strike a balance between religious observance and medical guidance. While guidelines from groups such as the Diabetes and Ramadan International Alliance and the International Diabetes Federation provide helpful suggestions, GPs are responsible for ensuring patient safety during Ramadan, particularly in areas where access to diabetes specialists is restricted. GPs are essential in managing diabetes-related issues before and throughout Ramadan, as well as in providing organized education and increasing awareness. A GP's primary responsibility at this time is to oversee the timely referral of high-risk patients and to effectively communicate with patients. To increase public awareness and support for diabetes care during Ramadan, it is also advised to work with religious leaders and make use of social media channels. It is ultimately in line with medical and religious beliefs to prioritize diabetes treatment during Ramadan, emphasizing the critical role general practitioners play in preserving the health and well-being of diabetics who are fasting.

## Editorial

The holy month of Ramadan is observed with fasting throughout the world by the Muslim community. Muslims abstain from all kinds of consumption (food, beverages, cigarettes, medications) from dawn to sunset [1]. About 2 billion Muslims throughout the world keep a fast during the holy month of Ramadan each year [2]. The population-based Epidemiology of Diabetes and Ramadan 1422/2001 (EPIDIAR) study demonstrated that among 12,243 people with diabetes from 13 Islamic countries, 43% of patients with type 1 diabetes and 79% of patients with type 2 diabetes fast during Ramadan [3]. In Pakistan, about 96.4% of the population is Muslim and reportedly 73% of diabetic patients observe Ramadan fasting for an average of 20-25 days, including both type 1 and type 2 diabetics [4].

The duration of fasting can range up to 18 hours in summer and the northern latitudes. These prolonged hours of fasting dramatically affect the eating and sleep patterns and lifestyles of patients with diabetes mellitus. The food intake becomes nocturnal and is characterized by huge fast-breaking meals in the evening and just before dawn. The sleeping intervals are shortened and the drug schedule during the daytime is changed because of fasting [5]. Because of these changes, patients with type 1 and type 2 diabetes mellitus are prone to develop acute and potentially life-threatening complications like hypoglycemia, hyperglycemia with dehydration, diabetic ketoacidosis, hyperosmolar hyperglycemic states, and increased risk of thrombosis in vital organs like the brain, heart, kidneys, and retina.

Taking all these risks into account, it is obvious why religious regulations, as well as medical recommendations, allow exemption from fasting for some people with diabetes. However, for many such patients, fasting is a deeply spiritual experience, and they will insist on taking part, perhaps unaware of the risks they are taking. Here comes the important role of physicians who should approach this issue with great sensitivity. Two main issues need to be solved by caring physicians. First, when to advise against fasting? Second, what should be the optimal therapeutic regimen for those who are allowed to fast to prevent complications and make Ramadan fasting safer for their patients?

Structured education interventions by scientific societies like the International Diabetes Federation, the Diabetes and Ramadan (IDF-DAR) International Alliance, and the National Institute for Health and Clinical Excellence (NICE) have made guidelines in association with religious bodies. These guidelines provide real-world recommendations to both diabetic patients who wish to fast and to the Health Care Providers (HCPs) who provide care. It is the utmost duty of the HCPs and especially the GPs to be fully aware of and updated with these guidelines to make this spiritual and religious experience as safe as possible.

However, most GPs believe that these guidelines and information could be better followed and delivered by physician experts in the field of diabetes and endocrinology. But keeping in view the limited number of diabetes experts and the huge number of diabetic patients in our country, Pakistan being third on the list with the greatest number of diabetic patients worldwide, it is not possible to deliver expert care to every patient. Therefore, the importance of GP's awareness of Ramadan fasting and diabetes care is paramount. Moreover, most of our poor diabetic patients reside in far-flung villages with no access to specialists and rely solely on the GPs working in Basic Health Units (BHUs), Rural Health Centers (RHCs), or private clinics. This further puts the responsibility on the shoulders of the GPs to be fully aware and equipped with the essential knowledge of diabetes care during Ramadan.

Therefore, HCPs and especially GPs should consider the practical management aspects of diabetes care before the start of the fasting month. They should attend the seminars arranged by the diabetes care departments and should equip themselves with relevant information material like posters and leaflets containing information regarding Ramadan fasting for people with diabetes. This information material ought to be displayed at least three months before the start of Ramadan. Timely referral of poorly controlled diabetic patients before the start of Ramadan and management of patients with diabetes-related complications during the month of Ramadan should be mastered and a close liaison should be kept with the patients in case of any urgent help needed.

In conclusion, making diabetes care safer and more effective during Ramadan is the religious and humanistic obligation of all GPs throughout the world. Following national and international guidelines can prevent many drastic complications of diabetes during the holy month of Ramadan. The guidance and unwavering care offered by GPs can result in profound and enduring positive impacts on the lives of diabetic patients, potentially saving them from life-altering conditions or disabilities, with effects extending beyond the present into the future. Finally, a collective approach involving religious leaders, such as Imams, and social media can never be overlooked in providing awareness and necessary information for the huge number of diabetic patients before the start of Ramadan.
